# Sweet's syndrome in a patient with compound heterozygous mutations in the Mediterranean fever gene (MEFV)

**DOI:** 10.1186/1546-0096-13-S1-P103

**Published:** 2015-09-28

**Authors:** M Michelson, Chana Vinkler, Dorit Lev

**Affiliations:** 1 Wolfson Medical Center, Clnical Genetics, Holon, Israel; 2 Maccabi Health Sevice, Wolfson Medical Center, Holon, Israel

## 

Sweet's syndrome (SS) or acute febrile neutrophilic dermatosis is a rare disorder that often occurs in association with other systemic diseases.

The disorder is characterized by development of nonpruritic, painful erythematous plaques with pseudovesicles, occasional pustules and rare bullae. SS consists of a triad of erythematous plaques infiltrated by neutrophils in association with fever and leukocytosis. The pathological features of SS involve the dermis.

The condition presents in three clinical settings: classic (or idiopathic) SS, malignancy-associated SS and drug-induced SS syndrome.

The treatment of choice for SS are systemic corticosteroids, although colchicine and potassium iodide are also considered to be effective for SS.

We present an unusual recurrent course of SS in a 38 year old man who carries compound heterogous mutations in the MEFV gene.

A 38 year old, generally healthy man from Sephardic Jewish ancestry, had suddenly developed fever, malaise, artralgia and painful erythematous plaques with pustules and bullae on the anterior aspects of the upper extremities. Diagnostic evaluation included moderate leucocytosis, elevated erythrocyte sedimentation and C-reactive protein rate and normal liver functions. antistreptolysin O-titer. Blood cultures and tuberculosis test were negative. Chest radiography was normal.

The symptoms exacerbated despite treatment with systemic corticosterids.

Clinical improvement appeared after administration of colchicine.

Mutational analysis of the MEFV gene revealed compound heterozygous M694V and V726A mutations.

Familial Mediterranean fever (FMF) is an autosomal recessive disease characterized by recurrent attacks of fever with serosal inflammation. The FMF gene (MEFV) encodes the protein pyrin that plays an important role in modulating the innate immune response.

MEFV mutations have been identified primarily in patients from Mediterranean populations and in Israel the carrier state is as high as1: 5.

Sweet's syndrome has been described in a patient with classical FMF as a possible new cutaneous feature and has never been described as a presenting sign of FMF.

Although various skin lesions have been described with FMF, erysipelas-like erythema (ELE) has been reported the only pathognomonic cutaneous manifestation.

Our patient, carrying compound heterozygous mutations in MEFV presented with Sweet's syndrome.

We suggest, that SS skin lesions might be an only cutaneous presentation of FMF.

Written informated consent for publication of their clinical details was obtained from the patient/parent/guardian/relative of the patient.

